# The MarR-Type Regulator MalR Is Involved in Stress-Responsive Cell Envelope Remodeling in *Corynebacterium glutamicum*

**DOI:** 10.3389/fmicb.2019.01039

**Published:** 2019-05-21

**Authors:** Max Hünnefeld, Marcus Persicke, Jörn Kalinowski, Julia Frunzke

**Affiliations:** ^1^Institute of Bio- and Geosciences, IBG-1: Biotechnology, Forschungszentrum Jülich, Jülich, Germany; ^2^Center for Biotechnology, Bielefeld University, Bielefeld, Germany

**Keywords:** MarR-type regulator, *C. glutamicum*, cell envelope, stress response, antibiotics, cell wall

## Abstract

It is the enormous adaptive capacity of microorganisms, which is key to their competitive success in nature, but also challenges antibiotic treatment of human diseases. To deal with a diverse set of stresses, bacteria are able to reprogram gene expression using a wide variety of transcription factors. Here, we focused on the MarR-type regulator MalR conserved in the *Corynebacterineae*, including the prominent pathogens *Corynebacterium diphtheriae* and *Mycobacterium tuberculosis*. In several corynebacterial species, the *malR* gene forms an operon with a gene encoding a universal stress protein (*uspA*). Chromatin affinity purification and sequencing (ChAP-Seq) analysis revealed that MalR binds more than 60 target promoters in the *C. glutamicum* genome as well as in the large cryptic prophage CGP3. Overproduction of MalR caused severe growth defects and an elongated cell morphology. ChAP-Seq data combined with a global transcriptome analysis of the *malR* overexpression strain emphasized a central role of MalR in cell envelope remodeling in response to environmental stresses. For example, prominent MalR targets are involved in peptidoglycan biosynthesis and synthesis of branched-chain fatty acids. Phenotypic microarrays suggested an altered sensitivity of a Δ*malR* mutant toward several β-lactam antibiotics. Furthermore, we revealed MalR as a repressor of several prophage genes, suggesting that MalR may be involved in the control of stress-responsive induction of the large CGP3 element. In conclusion, our results emphasize MalR as a regulator involved in stress-responsive remodeling of the cell envelope of *C. glutamicum* and suggest a link between cell envelope stress and the control of phage gene expression.

## Introduction

In almost every natural habitat, a high number of microbial species coexist and compete for space and nutrients. Consequently, the exposure to bacteriostatic or bactericidal compounds (antibiotics) represents a routine challenge, which bacteria are facing in various ecological niches, and particularly during infection of a specific host (Chao and Levin, 1981; Peschel, 2002; Stubbendieck and Straight, 2016). MarR-type transcriptional regulators constitute a prominent family of transcription factors involved in the reprogramming of gene expression in response to stress conditions (Wilkinson and Grove, 2006; Deochand and Grove, 2017). Already decades ago, clinical isolates of *Escherichia coli* displaying a multiple antibiotic resistance phenotype where found to carry mutations in the *marR* locus (George and Levy, 1983) and subsequently drew considerable attention to this ubiquitously found class of regulators. Following studies then showed that *E. coli* MarR is a transcriptional repressor of genes conferring resistance toward different antibiotics, organic solvents and lipophilic, mainly phenolic compounds (Alekshun and Levy, 1999). In further studies, it was shown that MarR-type regulators are widely distributed among bacteria and archaea, likely representing an ancient regulator family which emerged before the evolutionary split of these domains more than three billion years ago (Pérez-Rueda and Collado-Vides, 2001; Pérez-Rueda et al., 2004). Overall, the regulatory responses modulated by MarR-type regulators were grouped into three general categories (Wilkinson and Grove, 2006), including (i) environmental stress responses (e.g., triggered by antibiotics) (Poole et al., 1996; Srikumar et al., 2000; Spory et al., 2002), (ii) regulation of virulence genes (Lee et al., 2003; Rouanet et al., 2004), and (iii) degradation of lipophilic (often aromatic) compounds (Providenti and Wyndham, 2001; Galán et al., 2003). The DNA-binding domain of MarR-family regulators is typically comprised of a winged helix-turn-helix domain, recognizing palindromes, or inverted repeats (Grove, 2013). In the classical scenario, the dissociation of the MarR dimer from its genetic target is triggered by ligand binding [e.g., antibiotics, salicylates, and lipophilic compounds (Kumarevel, 2012)], but examples also exist where the binding of ligands fosters the association to DNA targets (Egland and Harwood, 1999; Providenti and Wyndham, 2001).

The suborder of the *Corynebacterineae* covers several prominent pathogenic species, such as *Corynebacterium diphtheriae, Mycobacterium tuberculosis*, and *Mycobacterium leprae*, causing millions of deaths every year. Species of this suborder share a very similar and unique cell wall composition hampering antibiotic treatment (Ortalo-Magne et al., 1995; Daffé and Draper, 1997; Zuber et al., 2008; Mishra et al., 2011; Marrakchi et al., 2014). In addition to the peptidoglycan, cells are surrounded by an arabinogalactan zone topped by a lipid bilayer composed of long-chain α-alkyl, β-hydroxy fatty acids – the mycolic acids (Eggeling et al., 2008).

In this study, we have characterized the function of the MarR-type regulator MalR (Cg3315) of the non-pathogenic, Gram-positive model organism *C. glutamicum* (Kalinowski et al., 2003), which – in total – harbors nine MarR-type regulators (Brune et al., 2005). Further, the genome of *C. glutamicum* contains a large prophage element (CGP3), which was shown to be inducible by the cellular SOS response (Nanda et al., 2014), or excises spontaneously in a small fraction of wild type cells (Frunzke et al., 2008; Helfrich et al., 2015).

*Corynebacterium glutamicum* MalR was previously reported as a repressor of the *malE* gene, encoding the malic enzyme (Krause et al., 2012). Here, we performed a genome-wide profiling of MalR targets by combining ChAP-Seq and a comparative transcriptomics approach. As revealed by phenotypic microarrays, a mutant lacking the *malR* gene displayed an impaired resistance toward different β-lactam antibiotics. The majority of former studies focused on a very distinct operon or small regulon controlled by MarR-type regulators. The present study provides – for the first time – a comprehensive insight into the complex regulon of MalR, which is involved in the remodeling of the cell envelope in response to stress conditions. Interestingly, our data also suggest a role of MalR in the control of the large cryptic prophage element CGP3 and thereby demonstrate a complex regulatory interaction between the host and horizontally acquired elements.

## Results

### The MarR-Type Regulator MalR Is Conserved in *Corynebacteria* and Mycobacteria

The MarR-type regulator MalR (Cg3315) was previously described as a repressor of the malic enzyme gene in *C. glutamicum* (Krause et al., 2012). Sequence analysis revealed that MalR is conserved in several coryne- and mycobacterial species, also including the prominent pathogens *C. diphtheriae* (57% sequence identity) and *M. leprae* (40% sequence identity). Simulated secondary structures of MalR using Phyre^2^ disclosed a high similarity to the secondary structure of MarR from *E. coli* consisting of six α-helices surrounding two β-sheets (Alekshun et al., 2001), although the amino acid sequence identity is only 22% (Supplementary Figure S1).

**FIGURE 1:**
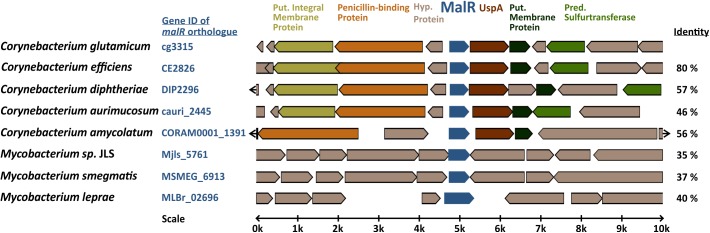
Genomic organization of the *malR* gene in coryne- and mycobacterial species. Amino acid sequence identity to the *C. glutamicum* MalR ortholog is given in the right column. The genomic context of *C. glutamicum malR* was extracted from microbesonline (http://microbesonline.org).

In the genome of *C. glutamicum* ATCC 13032, *malR* is organized in an operon with a gene encoding a universal stress protein (*uspA*) and is divergently located to a small hypothetical protein, followed by an operon coding for a penicillin-binding protein and two putative membrane proteins (Figure 1; Pfeifer-Sancar et al., 2013). This genomic ensemble emphasizes a role of MalR in global stress responses and potentially cell envelope-related functions. The genome-wide analysis of MalR target genes and its physiological impact is the aim of the present study.

### Genome-Wide Profiling of MalR Target Genes

**FIGURE 2:**
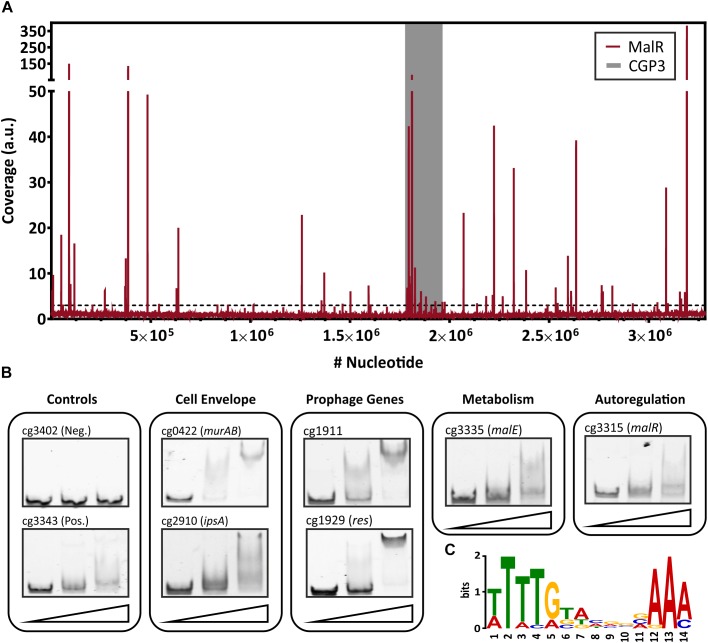
Genome-wide binding profile and *in vitro* verification of binding sites of MalR. **(A)** ChAP-Seq experiments revealed 66 distinct binding peaks of MalR in the genome of *C. glutamicum* ATCC 13032. For ChAP-Seq analysis, a strain containing a genomically encoded Strep-tagged variant of MalR was grown in CGXII medium containing 2% (w/v) glucose for 5 h and treated as described previously (Pfeifer et al., 2016). The coverage of MalR-bound DNA regions (red) was normalized using a rolling mean with a step size of 10 bp and a window size of 50 bp. The gray bar marks the CGP3 prophage region inside of the genome of *C. glutamicum*. Overall, 66 peaks with coverages higher than threefold mean coverage were detected. **(B)** Electrophoretic mobility shift assays (EMSAs) were performed to verify binding of MalR to promoter regions identified via ChAP-Seq. Therefore, 100 bp DNA fragments (50 bp up- and downstream the peak maximum) were incubated without protein (first lane), with 3 molar excess (228 nM, second lane), and 10 molar excess of purified MalR (760 nM, third lane). A complete overview on all tested fragment is shown in Supplementary Figure S2. **(C)** DNA sequences of 16 binding sites that were verified using EMSAs were used to deduce a possible binding motif of MalR with the online tool MEME-ChIP (Bailey et al., 2009). A motif based on all peaks obtained by ChAP-Seq, as well as the distribution of the motifs within the uploaded sequences, are shown in Supplementary Figure S3.

In order to identify target genes of MalR, ChAP-Seq analysis was performed and selected target promoters were subsequently verified using electrophoretic mobility shift assays (EMSA) (Figure 2). To produce MalR at physiological levels, a gene fusion was integrated at the *malR* locus into the *C. glutamicum* ATCC 13032 chromosome, encoding a C-terminally strep-tagged variant of MalR. Cells were grown in CGXII minimal medium with 2% glucose and harvested in the mid-exponential phase. The sequencing of DNA bound to MalR under the chosen conditions revealed 66 binding regions in total (Figure 2A and Supplementary Table S1). Remarkably, 13 target regions of MalR were found inside the cryptic prophage element CGP3, showing a local maximum in the region cg1895–cg1950 (8 peaks). Besides several genes of unknown function, MalR bound to promoter regions of genes involved in cell envelope biosynthesis, including *embC* (Alderwick et al., 2005), the *murAB* operon (Alderwick et al., 2015) and *ipsA* (Baumgart et al., 2013). It further associates to promoter regions of genes encoding proteins involved in transport mechanisms, such as *oppA*, cg1454, cg2256, and cg2340.

Conspicuously, 16 peaks were detected in the promoter region of genes coding for (putative) secreted proteins. Consistent with the report of Krause et al. (2012), also binding to the promoter region of *malE* was confirmed by our study. Furthermore, a significant binding peak was observed in the own promoter region of the *malR*-*uspA* operon, indicating an auto regulation of *malR* expression. In summary, this ChAP-Seq analysis revealed a global role of MalR in the regulation of genes involved in cell envelope-related functions and suggested a regulatory interaction of MalR with the large prophage CGP3.

To validate the obtained binding profile of MalR, EMSAs were performed using different promoter regions identified by ChAP-seq analysis (Figure 2B). Except one potential target promoter (cg2962), every tested candidate could be verified using this *in vitro* approach (Supplementary Figure S2). As a negative control, the promoter region of cg3402 (a putative copper chaperone) was used. Here, no shift was detectable. *In vitro*, different migration patterns were observed for the tested MalR targets, which likely reflect differences in binding affinities and/or the presence of multiple DNA motifs. Furthermore, in some cases, additional factors may contribute to *in vivo* MalR-DNA association (e.g., in the case of cg2962).

Using the 66 MalR peak sequences, a putative binding motif of MalR was deduced using the online tool MEME-ChIP (Bailey et al., 2009). This tool predicted a very AT-rich palindromic binding motif found in all peaks (motif and distribution in Supplementary Figure S3), which is very similar to the motif found in MalR targets verified with EMSAs (Figure 2C).

### Overproduction of MalR Causes Severe Growth Defects

**FIGURE 3:**
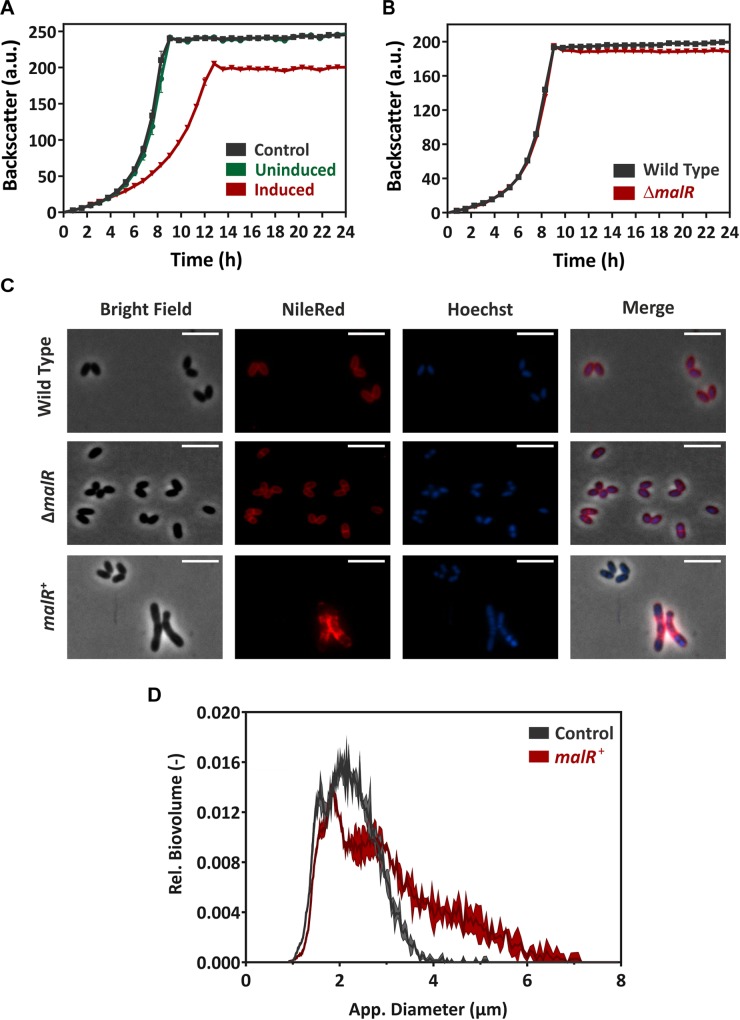
MalR overproduction causes severe growth defects of *C. glutamicum*. **(A)** Comparative growth experiment of *C. glutamicum* ATCC 13032 carrying the empty vector pEKEx2 and strain *C. glutamicum*/pEKEx2-*malR* overexpressing the *malR* gene. Cells were cultivated in CGXII minimal medium containing 2% (w/v) glucose with (“induced”) and without (“uninduced”) 25 μM IPTG using a microbioreactor cultivation system. **(B)** Growth of *C. glutamicum* ATCC 13032 in comparison with the strain lacking the *malR* gene. **(C)** For microscopic analysis, cells were grown in CGXII medium for 24 h at 30°C. Shown are wild-type *C. glutamicum* ATCC 13032 cells (row 1), *C. glutamicum* Δ*malR* cells (row 2), and *C. glutamicum* ATCC 13032 cells carrying the over expression vector pEKEx2-*malR* (row 3). The expression of *malR* is induced by the addition of 100 μM IPTG. Lipid components of the cell membrane were stained with Nile red (red); DNA was stained with Hoechst 33342 (blue). The scale bars represent 5 μm. Further microscopic pictures of cells overproducing MalR are shown in Supplementary Figure S5. **(D)** To quantify the number of cells with an altered morphology, cell counts and biovolume were analyzed using a MultiSizer 3 particle counter (Beckman Coulter, Brea, CA, United States) equipped with a 30 μm capillary in volumentric control mode. Cells were grown as described for **(C)**.

In a next step, we compared the growth of the *C. glutamicum* wild type with a *malR* deletion strain (Δ*malR*) and a strain overexpressing the *malR* gene under control of the IPTG-inducible P*_tac_* promoter (Figure 3). In fact, overexpression of *malR* caused a severe growth defect of *C. glutamicum* grown on CGXII minimal medium with 2% glucose (Figure 3A), whereas the deletion mutant had only a minor impact on the growth rate compared to the wild type strain under the tested conditions (Figure 3B).

Fluorescence microscopy of cells stained with NileRed (lipid components) and Hoechst 33342 (DNA) revealed a heterogeneous morphology of cells overexpressing the *malR* gene. Among cells with wild type cell shape, several cells displayed a significantly elongated cell morphology upon *malR* overexpression (Figure 3C). The deletion mutant, however, was indistinguishable from the wild type strain (Figure 3C). Furthermore, overexpression of *malR* resulted in an uneven distribution of the lipid fraction as revealed by Nile red staining. The cloudy and heterogeneous distribution of the Hoechst strain also pointed toward problems regarding nucleoid condensation and segregation in the strain overexpressing *malR*. In order to quantify the observed heterogeneous morphology of cells, culture samples were further analyzed using a MultiSizer 3 particle counter (Figure 3D). These data show a clear shift of the cells toward an increased cell volume.

### The Impact of Altered MalR Levels on Transcription

**FIGURE 4:**
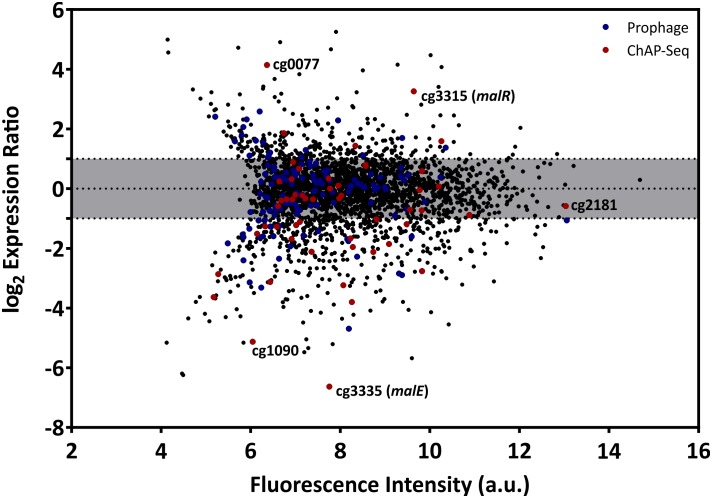
Overexpression of the *malR* gene causes global changes in the *C. glutamicum* transcriptome. Comparative transcriptome analysis of *C. glutamicum* ATCC 13032 cells carrying the overexpression vector pEKEx2-*malR* and a strain carrying the empty plasmid. Cells were cultivated in CGXII glucose minimal medium and harvested in the early exponential growth phase. Shown is an MA-plot where the log2 of the expression ratio is plotted against the fluorescence intensity of the single spots. Red dots indicate genes that were bound by MalR in the ChAP-Seq experiment (Figure 2).

The multitude of MalR-bound regions identified by ChAP-Seq analysis and the severe morphological changes caused by overexpression of *malR* already suggest a significant impact of MalR on the transcriptomic landscape of *C. glutamicum*. In the following research, we performed a comparative transcriptome analysis of the wild type, containing the empty vector pEKEx2, and the *malR* overexpressing strain, using DNA microarrays. For this purpose, both strains were grown in CGXII minimal medium and harvested in the early exponential phase. Additionally, we verified the obtained data using qRT-PCR with some selected samples (Supplementary Figure S4). As illustrated in Figure 4, *malR* overexpression resulted in massive changes in the global transcriptome when compared to the wild type. Overall, 170 genes showed a more than fivefold altered mRNA level (*p*-value < 0.05). A complete overview on the transcriptome analysis is provided in Supplementary Table S2. In contrast, deletion of the *malR* gene had only a minor impact under the tested conditions (Supplementary Table S2).

Considering the impact of increased MalR levels on growth and cell morphology, a majority of these effects are likely due to the result of secondary effects. To focus on primary targets of MalR, we analyzed the impact on the expression of genes whose promoter was directly bound by MalR as found *via* ChAP-Seq analysis (selection shown in Table 1; for a complete overview see Supplementary Tables S1, S2). In fact, many of the direct target genes of MalR revealed an altered mRNA level due to the overexpression of *malR*. In several cases, however, the effect was rather minor, which may be due to the chosen growth conditions lacking a specific effector molecule of the regulator. Furthermore, the majority of target genes is likely controlled by several regulatory systems affecting the transcriptional output.

**Table 1:** Genes bound by MalR with altered expression due to an overexpression of *malR.*

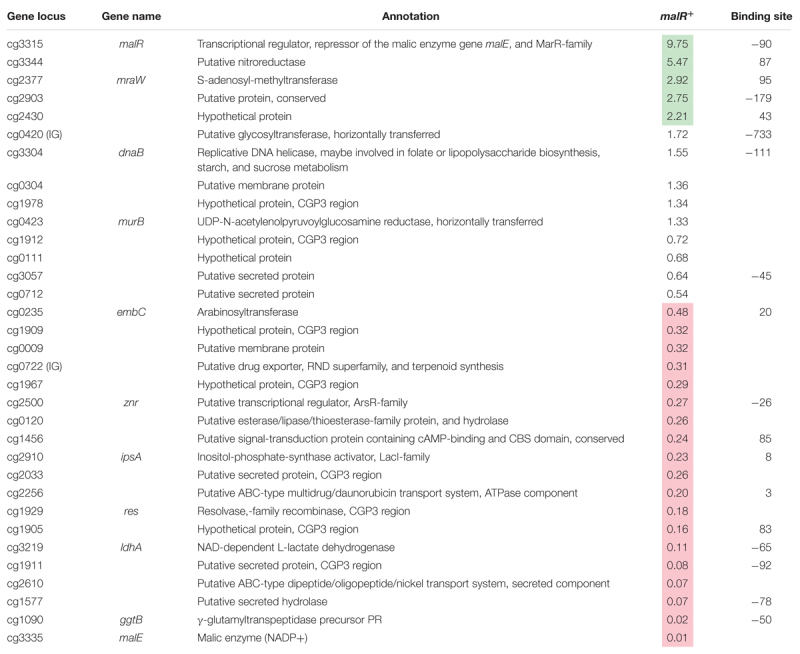

*Extraction of data shown in Figure 2, 4. The binding site is calculated from TSS (Pfeifer-Sancar et al., 2013) and the maximum peak position. *malR^+^* indicates the fold-change of the mRNA caused by an overexpression of *malR* (*p*-values < 0.05). IG, intragenic binding.*

Remarkably, many genes encoding proteins involved in cell envelope biosynthesis or remodeling were affected by *malR* overexpression. For example, the *ipsA* gene, encoding a LacI-type regulator, showed about a fivefold downregulation in the *malR* overexpression strain. IpsA was previously described as an important regulator modulating the synthesis of inositol-derived lipids in the cell wall of *C. glutamicum* (Baumgart et al., 2013). Cells lacking *ipsA* revealed an elongated cell morphology with an affected growth. These findings are in line with the phenotype of the MalR overproducing strain (Figure 3C). Among the genes repressed by MalR is the *embC* gene, encoding an arabinosyltransferase involved in arabinan biosynthesis (Belanger et al., 1996; Alderwick et al., 2005), and several (secreted) membrane proteins of unknown function (cg0623, cg0636, cg0879, cg0952, cg1578, cg1910, and cg3322). The most distinct downregulation was observed for the *malE* gene, encoding the malic enzyme which catalyzes the decarboxylation of malate to pyruvate. Pyruvate itself is a precursor for acetyl-CoA synthesis, which is also required for fatty acid synthesis.

Among the genes showing a slightly increased mRNA level in response to *malR* expression, we also found the *ilvA* gene, encoding a threonine-dehydratase that is necessary for the production of isoleucine (Sharma et al., 2016). Isoleucine is a branched chain amino acid and, together with acetyl-CoA, an important precursor for the generation of branched chain fatty acids, which are part of the bacterial cell membrane. Furthermore, the *oppA* gene was slightly upregulated, which codes for an oligopeptide permease required for the modulation of cell-wall associated lipids as well as mycolic acids (Flores-Valdez et al., 2009). The expression level of methyltransferase *mraW* was about threefold increased. In *E. coli*, MraW was described to play an important role during cell division (Carrión et al., 1999).

Among the direct targets of MalR, we also found the gene *murB*, which is involved in the synthesis of peptidoglycan building blocks by converting UDP-N-acetylglucosamine partially to UDP-N-acetylmuramic acid (Burkovski, 2013). However, its mRNA level was almost unaffected by *malR* overexpression, suggesting that further regulatory components are involved in the control of the *murAB* operon. Altogether, ChAP-Seq analysis, the impact of MalR on cell morphology and this transcriptomic study strongly emphasize an important role of MalR in the remodeling of the cell envelope.

### MalR Affects the Cell Surface Structure of *C. glutamicum*

**FIGURE 5:**
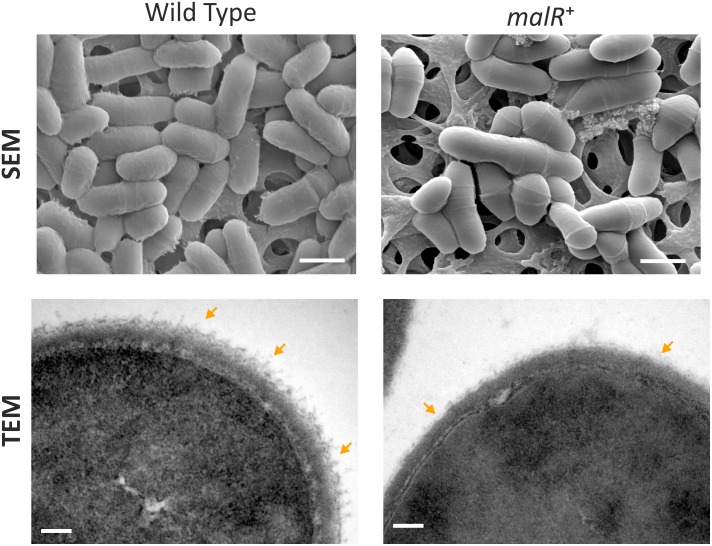
*C. glutamicum* cells overproducing MalR show an altered cell surface structure. Shown are SEM and TEM microscopy pictures of wild type cells and cells overproducing the MalR protein. SEM pictures are 15000 x magnified; TEM pictures 167000×. For microscopic analysis, cells were cultivated in CGXII minimal medium containing 2% (w/v) glucose and *malR* expression was induced by adding 100 μM IPTG. Cells were fixed using 3% Glutaraldehyde in Sörensen phosphate buffer.

Considering the impact of MalR on cell morphology (Figure 3C), we analyzed cells overproducing MalR using transmission electron microscopy (TEM) and scanning electron microscopy (SEM) (Figure 5). Both approaches suggested differences in the cell surface structure. While wild type cells show a rather homogenous distribution in size, the strain overexpressing *malR* displayed an elongated cell morphology and significant heterogeneity, with regard to cell size (Figure 3C, 5). Moreover, the overall cell surface structure appeared smoother. The fuzzy structure observed by TEM is also typical for the outer layer of the mycobacterial envelope (Zuber et al., 2008). This electron-dense layer is supposed to consist of a protein-carbohydrate matrix with only a few lipids (Daffé and Draper, 1997).

### MalR Confers Increased Resistance Toward Cephalosporin Antibiotics

**FIGURE 6:**
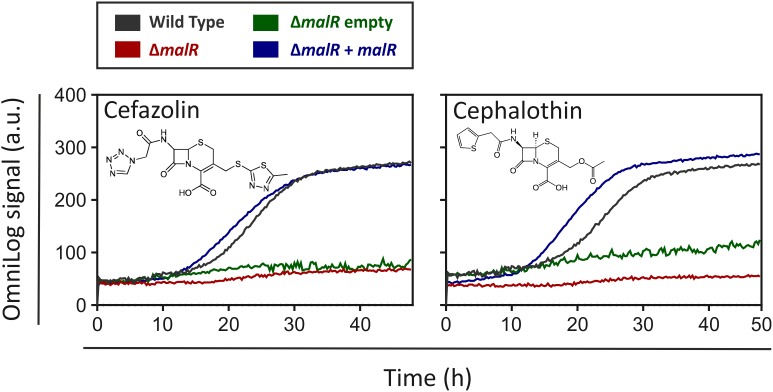
The Δ*malR* mutant strain shows a higher sensitivity toward cephalosporins in phenotypic microarrays. An OmniLog System from Biolog (Hayward, CA, United States) was used to perform phenotypic microarrays with wild type *C. glutamicum* ATCC 13032 as well as the *malR* deletion strain. The experiments were conducted like described in the protocol of the manufacturer (Bochner et al., 2001). One group of compounds that led to a difference in respiration of wild type cells and Δ*malR* cells were the cephalosporins (Cefazolin: 0.58 μg/ml; Cephalothin: 6 μg/ml).

For a better understanding of the physiological impact of MalR, we performed phenotype microarrays of the wild type and the Δ*malR* mutant using a Biolog system. Here, we focused on the plates PM1 and PM2A (carbon sources), PM4 (phosphorus and sulfur sources), PM9 (osmolytes), PM10 (pH), and PM11-PM13 (antibiotics). The only additives that led to a different behavior between the wild type and the *malR* deletion strain were different antibiotics. To be precise, different β-lactams, tetracyclines and other examples of different substance classes revealed an altered metabolic activity of the Δ*malR* mutant (Supplementary Table S4). Figure 6 shows two examples, emphasizing a significantly increased sensitivity of the mutant toward different cephalosporins. Compared to the wild type, which was able to tolerate moderate levels of the antibiotics cefazolin (0.58 μg/ml) and cephalothin (6 μg/ml), the Δ*malR* strain was significantly affected, and did not restore metabolic activity within 40 h under the tested conditions. This phenotype was successfully complemented by plasmid-encoded MalR (*C. glutamicum* Δ*malR*/pEKEx2-*malR*) when the strain was compared to the empty vector control (*C. glutamicum*/pEKEx2). An overview of all tested plates is provided in Supplementary Figure S6. Remarkably, some changes in the antibiotic tolerance profile could also be attributed to the presence of kanamycin used as selection marker of the respective plasmid. An additional growth experiment with the *malR* deficient strain, harboring either the empty vector or the plasmid pEKEx2-*malR*, was performed to verify the complementation (Supplementary Figure S7).

### MalR Counteracts SOS-Dependent Induction of the CGP3 Prophage

**FIGURE 7:**
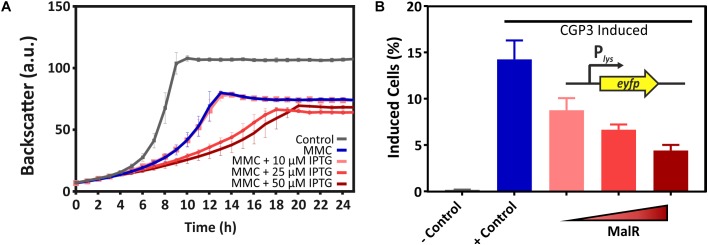
Overexpression of *malR* counteracted SOS-dependent prophage induction. *C. glutamicum* ATCC 13032::P*_lys_*-*eyfp* cells containing the plasmid pEKEx2-*malR* were cultivated in CGXII minimal medium containing 2% (w/v) glucose (+25 μM Kanamycin) using a microbioreactor cultivation system. CGP3 induction was triggered by addition of 600 nM mitomycin C (MMC). For the negative control, no MMC was added. Additionally, different concentrations of IPTG (0–50 μM) were used to induce expression of *malR*. Both, MMC and IPTG, were added directly at the beginning of the cultivation (0 h). Under the applied conditions, we analyzed the strain with regard to growth **(A)**. Furthermore, the fraction of CGP3-induced cells was assessed in the stationary phase (after ∼25 h of cultivation) using an Accuri C6 flow cytometer **(B)**.

Due to several binding sites inside the CGP3 region, an impact of MalR on the inducibility of this large cryptic prophage was the focus of further experiments. For this purpose, the reporter strain *C. glutamicum* ATCC 13032::P*_lys_*-*eyfp* carrying the *malR* overexpression plasmid pEKEx2-*malR* was used. The P*_lys_*-*eyfp* reporter enables the visualization of prophage induction within single cells by the production of the fluorescent protein eYFP (Helfrich et al., 2015). In the following, we triggered an induction of the cellular SOS response by the addition of the DNA-damaging agent mitomycin C (MMC) and monitored its impact on CGP3 induction. The MalR level was modulated by adding increasing amounts of IPTG (10, 25, and 50 μM). Remarkably, the fraction of CGP3 induced cells significantly declined in response to *malR* overexpression (Figure 7B). Also, the growth of the strains was severely affected upon addition of MMC and IPTG (Figure 7A). The dose responsive behavior of prophage induction in response to *malR* overexpression suggested that MalR counteracts prophage excision under the tested conditions.

## Discussion

With this study, we provided comprehensive insights into the complex regulon of the MarR-type regulator MalR in *C. glutamicum*. In the last few decades, members of this regulator family were rewarded with considerable attention as some MarR proteins were shown to contribute to a so-called multiple antibiotic resistance phenotype (Alekshun and Levy, 1999; Deochand and Grove, 2017). In several cases, MarR-type regulators were described to control a small set of target genes, often located in the same operon or in divergent orientation to the regulator gene on the chromosome (Alekshun and Levy, 1999). The resulting phenotype of increased antibiotic resistance was previously proposed to be a result of decreased influx and increased efflux of the toxic compound. In this study, we now provided a genome scale profiling of MalR binding and identified more than 60 promoter regions bound by this regulator. A combination of ChAP-Seq analysis and comparative transcriptomics emphasizes MalR as a global regulator of stress-responsive remodeling of the cell envelope. Remarkably, MalR is conserved in several species of the genera *Corynebacterium* and *Mycobacterium*, also including prominent pathogens like *C. diphtheriae* and *M. leprae*. Conspicuously, the genomic organization of the *malR* locus in *C. diphtheriae* is almost identical to *C. glutamicum*, where *malR* forms an operon with a gene (*uspA*) encoding *uspA* (Kalinowski et al., 2003). The superfamily of Usp proteins comprises a large group of conserved proteins that can be found in all domains of life (Kvint et al., 2003). In *M. bovis* BCG, the tuberculosis vaccine strain, overexpression of a particular Usp led to an increased susceptibility of the cells toward the anti-tuberculosis drug isoniazid (Hu et al., 2015). This conserved genomic organization of the *malR* locus in these species is in favor with a similar role of *C. diphtheriae* MalR in cell envelope remodeling and antibiotic resistance in this important human pathogen.

The effector molecule of MalR has not yet been identified, but based on our findings, we can speculate that MalR binding is affected by one or several antibiotics and/or lipophilic compounds causing cell envelope stress. Interestingly, the results of our phenotypic microarrays revealed a clear difference in respiration of the wild type and the *malR* deficient strain when cephalosporins of the first generation (Cephalothin, Cefazolin) or the second generation (Cefuroxime) were added to the medium. A link between cephalosporins and MalR is provided by cg3313, encoding a penicillin-binding protein, which is located in divergent orientation to the *malR-uspA* operon (Figure 1). Penicillin-binding proteins are involved in the cell wall synthesis, to be more precise in the peptidoglycan synthesis (Valbuena et al., 2007), and are in general targets of different β-lactam antibiotics including cephalosporins (Fontana et al., 2000). Upon overexpression of *malR*, four (out of nine in total) genes encoding penicillin-binding proteins showed a more than twofold change in transcription (Supplementary Table S2). Nevertheless, only the first and second generation cephalosporins showed an antibiotic impact on the tested strains. Another antibiotic of the third generation of cephalosporins (Ceftriaxone) did not have any effect on the respiration of both strains. Considering the history of cephalosporin development, the described effect can be elucidated: while first and second-generation cephalosporins were mainly active against Gram-positive bacteria, with the third generation an R1 methoxy substitution changed the specificity more toward Gram-negative bacteria, which simultaneously reduced the effect of those cephalosporins toward Gram-positive penicillin-binding proteins (Craig and Andes, 2015).

The genome of *C. glutamicum* comprises in total nine MarR-type transcriptional regulators; four of which were already characterized in former studies. Except for MalR, no impact on antibiotic resistance phenotype for any of the previously studied examples has been reported so far. RosR, which constitutes a hydrogen peroxide sensitive regulator, was shown to play an important role in the oxidative stress response of *C. glutamicum* (Bussmann et al., 2010). The MarR-type regulator PhdR was shown to act as a repressor of the *phd* gene cluster important for phenylpropanoid utilization in *C. glutamicum*. Here, phenylpropanoids or their degradation intermediates were shown to cause dissociation of PhdR and de-repression of the respective target operon (Kallscheuer et al., 2016). Finally, the isoprenoid pyrophosphate-dependent regulator CrtR was recently described as being involved in the regulation of carotenoid biosynthesis and thus represents an example for a rather specialized MarR-type regulator (Henke et al., 2017). MalR itself was firstly reported by Krause et al. as a repressor of the *malE* gene, encoding an NADP^+^ - dependent malic enzyme in *C. glutamicum* (Krause et al., 2012). This role is supported by our study, where *malE* was among the genes most affected by MalR overexpression. MalE catalyzes the decarboxylation of malate to pyruvate while generating NADPH (Gourdon et al., 2000). Pyruvate, a precursor for acetyl-CoA synthesis, as well as NADPH as a reducing agent, are required for fatty acid biosynthesis (Cronan and Thomas, 2009). For different oleaginous microorganisms, it is known that malic enzymes play a crucial role in lipid generation (Li et al., 2013; Zhang et al., 2013; Ratledge, 2014). For example, two malic enzymes were recently shown to be important for triacylglycerol and antibiotic production in *Streptomyces coelicolor* (Rodriguez et al., 2012).

Genome-wide profiling of MalR-bound DNA using ChAP-Seq analysis unraveled more than 60 direct target genes and operons in addition to *malE*. Thus, the global impact of MalR ranges from peptidoglycan biosynthesis [*murA*-*murB* (Burkovski, 2013)] to the synthesis of arabinogalactan [*embC* (Alderwick et al., 2005)], and cell wall associated lipids and mycolic acids (e.g., via *oppA* and *ipsA*). For example, the *oppA* gene codes for an oligopeptide permease, which was further characterized in *M. tuberculosis* (Flores-Valdez et al., 2009). Flores-Valdez and others could show that OppA is required for the modulation of cell-wall associated lipids as well as mycolic acids. The LacI-type regulator IpsA, which is itself repressed by MalR, was previously shown to trigger the expression of the *ino1* gene encoding the inositol phosphate synthase. Deletion of *ipsA* resulted in a severe decrease of inositol-derived lipids and an abolished mycothiol biosynthesis (Baumgart et al., 2013). Remarkably, a Δ*ipsA* mutant features a similar cell morphology as *malR* overexpression, suggesting that some phenotypic effects may be indirectly resulting from reduced IpsA levels (Figure 3C).

A role in cell envelope remodeling appears to be a common theme in the family of MarR regulators. For example, the MarR-type regulator SlyA was shown to control several targets impacting cell envelope composition, some of which have direct implications on the resistance of *Salmonella enterica* toward antibiotics (e.g., polymyxins) or virulence (Navarre et al., 2005). The regulator Rv1404 from *M. tuberculosis* was also shown to contribute to an adaptation of the cells to the host environment by enhancing the acid-tolerance of this bacterium (Healy et al., 2016). Altogether, these examples emphasize different roles of MarR-type regulators. Whereas some proteins appear to conduct very distinct regulatory functions, like the control of a certain catabolic gene cluster, others (like MalR or SlyA) act as global regulators orchestrating complex adaptive strategies in response to environmental stresses.

A further prominent target of MalR appeared to be the cryptic prophage element CGP3. MalR bound to 13 regions within the CGP3 element overall and overexpression of the *malR* gene counteracted CGP3 induction upon addition of the SOS inducing antibiotic mitomycin C. So far, little is known about the role of MarR-type regulators in the control of horizontally acquired elements. In *Bacillus subtilis*, a *pamR* deficient strain displayed altered expression of prophage genes, however the precise regulatory impact remained unclear (De San Eustaquio-Campillo et al., 2017). Another example is depicted by RovA, which is a MarR-type transcription factor in *E. coli, Yersinia pseudotuberculosis* and its homolog SlyA from *Salmonella* (Navarre et al., 2007). Former studies revealed that RovA and SlyA act as countersilencers of H-NS target promoters controlling genes that impact virulence in *Yersinia and Salmonella species* (Heroven et al., 2004; Navarre et al., 2005, 2006; Ellison and Miller, 2006). Remarkably, this regulatory plasticity was also the subject of a recent study by Will et al., highlighting the ability of MarR-type regulators to act as counter-silencers of horizontally acquired genes. This feature of this regulator family has significantly contributed to the evolution of *Enterobacteriaceae* by horizontal gene transfer (Will et al., 2019). We previously reported on the xenogeneic silencer CgpS, which plays a crucial role in the silencing of the CGP3 island by binding to AT-rich DNA (Pfeifer et al., 2016, 2019). MalR itself binds to an AT-rich palindromic motif whose composition, of course, increases the likelihood of an overrepresentation in horizontally acquired regions. However, the precise regulatory impact of MalR on CGP3 activation and a potential role in counter-silencing CgpS activity remains unclear. The observed reduction of CGP3 induction in response to *malR* overexpression speaks against a counter-silencing mechanism as reported for RovA. In contrast, the majority of phage targets appeared to be repressed by MalR. In physiological terms, a repressive function of MalR toward CGP3 genes could be overcome by the presence of an effector molecule leading to a dissociation of a putative MalR dimer. Therefore, we could speculate on a role of MalR in stress-responsive induction of the CGP3 prophage, which could literally leave the “sinking ship” when the life of its host is threatened by harsh environmental conditions.

With this study, we provide a comprehensive overview on the many targets controlled by MalR and suggest an important function of this MarR-type regulator in the coordinated control of genes with an impact on cell envelope composition. The relevance of this global response is reflected by the severely increased sensitivity of a *malR* mutant to several β-lactam antibiotics and is further supported by several other cases where members of this family contributed to enhanced antibiotic resistance (Poole et al., 1996; Alekshun and Levy, 1999; Lee et al., 2003; Navarre et al., 2005). We have gained a first glimpse on a complex adaptive response. However, many – if not most – of the MalR targets encode proteins of unknown function. For several others, only very limited data are available. So, many more studies are needed to understand the molecular principles behind this adaptive response. With the multitude of targets identified in this study, we provide a starting point for further studies aiming to enhance our understanding of bacterial adaptation to stress.

## Materials and Methods

### Bacterial Strains, Plasmids, and Growth Conditions

All bacterial strains and plasmids used in this work are listed in Table 2. For cloning and plasmid construction, the strain *E. coli* DH5α was used, whereas the strain *E. coli* BL21 was used for protein production. These strains were – unless stated otherwise – cultivated in lysogeny broth [LB, (Sambrook and Russell, 2001)] containing 50 μg/ml Kanamycin. *Corynebacterium glutamicum* ATCC 13032 was used as a wild type strain (Kalinowski et al., 2003). All derived *C. glutamicum* strains were cultivated in brain heart infusion medium (BHI, Difco Laboratories, Detroit, MI, United States) or in CGXII minimal medium containing 2% (w/v) glucose (Keilhauer et al., 1993); if necessary 25 μg/ml Kanamycin was added. For the cultivation of *C. glutamicum*, a first pre-culture was inoculated with single colonies from agar plates, either directly after transformation or after a streak-out of glycerol cultures. These pre-cultures were conducted in 4.5 ml BHI medium in test tubes or – depending on the purpose – in 1 ml BHI medium in 96-well deep well plates (DWPs) at 30°C for 8 h. Afterward, cells were used for inoculating a second overnight pre-culture in CGXII medium containing 2% (w/v) glucose. This CGXII culture was used to inoculate a main culture in the same medium to an OD_600_ of 1.

**Table 2:** Strains and plasmids used in this work.

Strain or plasmid	Relevant characteristics	Source
***E. coli***		
DH5α	*supE44*Δ*lacU169* (φ80*lac*ZDM15) *hsdR17 recA1 endA1 gyrA96 thi-1 relA1*	Invitrogen
BL21 (DE3)	F- *ompT hsdS*_B_ (r_B_-, m_B_-) *gal dcm* (DE3)	Studier and Moffatt, 1986
***C. glutamicum***		
ATCC 13032	Biotin-auxotrophic wild type	Kinoshita et al., 2004
ATCC 13032 Δ*malR*	ATCC 13032 with an in-frame deletion of *malR* (cg3315)	This work
ATCC 13032::*malR*-*strep*	ATCC 13032 coding for a Strep-Tag fused C-terminally to MalR; integrated at the native locus	This work
ATCC 13032::P*_lys_*-*eyfp*	ATCC 13032 containing the prophage reporter P*_*lys*_*-*eyfp* integrated into the intergenic region of cg1121-cg1122	Helfrich et al., 2015
**Plasmids**		
pEKEx2	Kan^R^; expression vector with *lacI^q^*, P*_tac_* and pUC18 multiple cloning site	Eikmanns et al., 1991
pEKEx2-*malR*	Kan^R^; P*_tac_*, overexpression vector for *malR* (cg3315)	This study
pJC1	Kan^R^, Amp^R^, and *C. glutamicum* shuttle vector	Cremer et al., 1990
pJC1-P*_lys_*-‘lys-venus	Kan^R^, Amp^R^, and prophage reporter plasmid with the gene for the fluorescent protein Venus under control of the promoter of cg1974 (putative phage lysine)	(Pfeifer, unpublished)
pK19 *mob sacB*	Kan^R^; plasmid for allelic exchange in *C. glutamicum*; (pK18 *oriV_E.c._, sacB, lacZα*)	Schäfer et al., 1994
pK19-*malR*-C-*strep*	Kan^R^; plasmid for tagging *malR* (cg3315) genomically with a C-terminal Strep-tag	This study
pK19-Δ*malR*	Kan^R^; plasmid for the in-frame deletion of *malR* (cg3315)	This study
pET24b	Kan^R^; vector for overexpression of genes in E. coli, with optional C-terminal hexahistidine affinity tag (pBR322 oriVE.c. PT7 lacI)	Novagen
pET24b-*malR*-C-*strep*	Kan^R^; plasmid for overexpression of C-terminal Strep-tagged *malR*	This study


Growth experiments were conducted in the BioLector microbioreactor system (m2p labs, Baesweiler, Germany) (Kensy et al., 2009). Therefore, 750 μl CGXII medium containing 2% (w/v) glucose and the particular stated additives [e.g., Isopropyl β–D-1-thiogalactopyranoside (IPTG)] were inoculated to an OD_600_ of 1 in 48-well microtiter plates (Flowerplates, m2p labs) and cultivated for at least 24 h at 30°C and 1200 rpm shaking frequency. Fluorescence, as well as backscatter measurements, were taken every 15 min.

### Recombinant DNA Work and Construction of Chromosomal Insertions or Deletions

All standard laboratory methods (PCR, DNA restriction, Gibson Assembly) were performed according to standard protocols and manufacturer’s instructions (Sambrook and Russell, 2001; Gibson et al., 2009). The used oligonucleotides, as well as details regarding the plasmid construction are provided in the Supplementary Tables S3A,B.

For chromosomal integration or deletion, a two-step homologous recombination system based on the suicide vector pk19*mobsacB* was used (Schäfer et al., 1994; Niebisch and Bott, 2001). This vector contained 500 bps of each site flanking the targeted sequence in the genome of *C. glutamicum*.

### Chromatin Affinity Purification and Next Generation Sequencing (ChAP-Seq)

Pre-cultures for ChAP-Seq were conducted as described above. As a main-culture, *C. glutamicum* ATCC 13032::*malR-strep* was grown in CGXII medium containing 2% (w/v) glucose for 5 h at 30°C and 120 rpm shaking frequency. Cells were then harvested by centrifugation (10 min at 11,325 × *g* and 4°C). Subsequently, the cells were washed once with CGXII medium without MOPS and resuspended in the same medium containing 1% (v/v) formaldehyde as a fixation agent and incubated at room temperature for 20 min. To stop this, glycine was added to a final concentration of 125 mM, followed by an additional 5 min incubation step at room temperature. To remove the remaining formaldehyde, the cells were washed twice with buffer A (100 mM Tris–HCl, pH 8.0, 1 mM EDTA), and afterward resuspended in 25 ml buffer A, containing *cOmplete* Protease Inhibitor (Roche, Basel, Switzerland) and 2.5 mg RNase A. The subsequent preparation of ChAP-seq samples (cell disruption via French press, sonification, and ultracentrifugation) was conducted as described by Pfeifer et al. (2016). Afterward, the supernatant was then purified using a 2-ml bed volume Strep-Tactin-Sepharose column (IBA, Göttingen, Germany), following the manufacturer’s protocol. The elution fractions were pooled, SDS was added to a final concentration of 1% (w/v), and incubated over night at 65°C, followed by a treatment with proteinase K (final concentration 400 mg ⋅ ml^-1^) for 3 h at 55°C. In a last step, the DNA of the samples was purified by phenol–chloroform extraction using Roti^^®^^-phenol/chloroform/isoamyl alcohol (Carl Roth, Karlsruhe, Germany), precipitated with ethanol and 0.3 M sodium acetate, washed with 70% (v/v) ethanol, dried and resuspended in 100 μl H_2_O. Further steps for the analysis of the gained DNA (sequencing, trimming, data analysis) were conducted as described by Pfeifer et al. (2016).

### Protein Purification

MalR with a C-terminal Strep-tag was heterologously produced in *E. coli* BL21 (DE3), transformed with pET24b-*malR*-C-strep. Cells were grown to an OD_600_ of 0.6 at 37°C as described above. Subsequently, the protein production was induced using 100 μM IPTG and the cultivation was continued for 5 h at 16°C. Cells were harvested by centrifugation for 15 min at 5300 × *g* and 4°C and the pellets were snap-frozen using liquid nitrogen. For cell disruption, the pellets were thawed on ice and resuspended in buffer A (50 mM Tris–HCl, 1mM EDTA, pH 8.0) containing *cOmplete* Protease Inhibitor (Roche, Basel, Switzerland). This cell suspension was then treated with a French pressure cell for three passages at 172 MPa. Cell debris was removed by centrifugation for 30 min at 5300 × *g* and 4°C and a subsequent ultracentrifugation for 1 h at 150,000 × *g* and 4°C. The tagged protein was then purified with a 1-ml bed volume Strep-Tactin-Sepharose column (IBA, Göttingen, Germany), following the manufacturer’s protocol.

### Electrophoretic Mobility Shift Assays

As part of the investigation of the binding properties of MalR and as an *in vitro* verification of the results obtained by ChAP-seq analysis, EMSAs were performed. Therefore, 100 bp DNA fragments, centering the peak maximum of each particular promoter region, were amplified using PCR (oligonucleotide sequences are given in the Supplementary Table S3C) and analyzed and purified using an agarose gel with subsequent gel extraction with the “PCR clean-up and Gel extraction” Kit from Macherey-Nagel (Düren, Germany). A total of 90 ng of DNA per lane was incubated with different molar excesses of purified MalR protein (threefold and 10-fold molar excess) for 30 min in bandshift-buffer [50 mM Tris–HCl, 5 mM MgCl_2_, 40 mM KCl, 5% (v/v) glycerol, pH 7.5]. Subsequently, samples were separated using a 10% native polyacrylamide gel electrophoresis as described previously (Pfeifer et al., 2016).

### Transcriptome Analysis Using DNA Microarrays

To analyze the global transcriptomic alterations triggered by an overexpression of *malR C. glutamicum* ATCC 13032 cells, harboring either the empty vector pEKEx2 or the overexpression plasmid pEKEx2-*malR*, were cultivated in CGXII medium containing 2% (w/v) glucose, and 100 μM IPTG as described previously. Subsequently, total RNA of these cultures was prepared using the RNeasy Mini Kit (QIAGEN, Hilden, Germany) following the manufacturers protocol. The cDNA labeling, and the DNA microarray analysis was performed as described previously (Donovan et al., 2015). The array data have been deposited in the GEO database^1^ under the accession number: GSE116655.

### Verification of the Transcriptomic Data Measuring mRNA Levels by Quantitative Real-Time PCR (qRT-PCR)

Preparation of total RNA from *C. glutamicum* cultures was carried out as described above. Measurement of differential gene expression was conducted using a qTower 2.2 (Analytik Jena, Jena, Germany) with the Luna^^®^^ Universal One-Step RT-qPCR Kit (New England Biolabs, Ipswitch, United States). Primer pairs used for the analysis are listed in Supplementary Table S3D. For all samples, 100 ng of total RNA were used as a template and all measurements were performed in biological as well as in technical duplicates. The Ct-values of the samples were obtained using qPCR-soft 3.1 (Analytik Jena). Subsequently, the relative transcriptional changes were calculated using the following equation:

$$\begin{array}{c} \Delta Ct=Ct(\mathrm{sample})-Ct(\mathrm{control})\\ \mathrm{Relative}\;\mathrm{transcriptional}\;\mathrm{change}=2^{-\mathrm{\Delta Ct}} \end{array}$$

### Fluorescence Microscopy and Staining

For microscopic analysis, cells were cultivated in CGXII medium containing 2% (w/v) glucose (as described above) and grown for 24 h at 30°C. Lipids were stained with Nile red and DNA was stained with Hoechst 33342 (Sigma-Aldrich, Munich, Germany). Therefore, 10 μl of the cell suspensions were centrifuged for 5 min at 8,000 × *g* and the pellet was resuspended in 500 μl PBS containing 100 ng/ml Hoechst 33342 and 250 ng/ml Nile red. The cells were incubated for 30 min at room temperature, and subsequently analyzed microscopically using an AxioImager M2 (Zeiss, Oberkochen, Germany) with a Zeiss AxioCam MRm camera and a Plan-Apochromat 100×, 1.40 Oil phase contrast oil-immersion objective. Fluorescence was measured using the 63 HE filter for Nile red fluorescence and the filter set 49 for Hoechst fluorescence.

The optimal exposure time for the different fluorescence images was determined with the automatic measurement option of the AxioVision Rel. 4.8 software (Carl Zeiss MicroImaging GmbH) and the pictures were analyzed with the same software.

### Cell Counting and Determination of the Cell Size

Cell counts and biovolume were analyzed using a MultiSizer 3 particle counter (Graham, 2003) equipped with a 30 μm capillary in volumetric control mode. For the measurement, cells were grown for 24 h as described above and afterward diluted to an OD_600_ ≤ 0.025 in CASYton buffer (Schärfe Systems, Reutlingen, Germany). Only particles sizing from 0.633 to 18 μm were analyzed.

### Phenotypic Analysis of *C. glutamicum* Δ*malR* and *C. glutamicum* ATCC 13032

Phenotypical characterization of the strains *C. glutamicum* Δ*malR* and *C. glutamicum* ATCC 13032 was performed using the Phenotypic MicroArrays from BIOLOG (Biolog Inc., Hayward, CA, United States). Both strains were compared regarding their respiratory activity in the presence of various carbon sources (PM1 and PM2), phosphorus and sulfur sources (PM4), different osmolytes (PM9), pH-values (PM10), and antibiotics (PM11, PM12, and PM13). Experimental setup was carried out as described in the BIOLOG protocol for analysis of *Bacillus subtilis* and other Gram-positive bacteria (Biolog Inc., Hayward, CA, United States). In short, both strains were grown overnight at 30°C on blood agar plates. Cells were inoculated in the different PM-media containing 1% (v/v) of the redox dye (dye mix F) to a turbidity of 81% transmittance. Afterward, each well of the PM-plates was filled with 100 μl of the corresponding inoculation medium. Phenotypic MicroArrays were analyzed using the OmniLog incubator (Biolog Inc., Hayward, CA, United States) at 30°C for 48 h with a measuring rate of 15 min. Data visualization was performed by the BIOLOG software OM_Pl_Par 1.20.02 for parametric analyses. For selected examples, GraphPad Prism 7 was used for visualization. An overview of all results is shown in Supplementary Figure S6.

### Flow Cytometric Analysis

The CGP3 prophage induction was assessed by flow cytometric analysis of a *C. glutamicum* strain, harboring a genomically integrated prophage reporter (ATCC 13032::P*_lys_*-*eyfp*), using a BD Accuri C6 flow cytometer (BD biosciences, Heidelberg, Germany). Cells were cultivated in CGXII medium containing 2% (w/v) glucose (as described above) and grown for 25 h at 30°C. As appropriate, different concentrations of IPTG (10, 25, and 50 μM), as well as 600 nM Mitomycin C, were used. Flow cytometric analysis was performed using a 488 nm laser and a 530/30 nm filter for measuring eYFP fluorescence. In total, 100,000 events were analyzed per sample and data was analyzed using BD Accuri C6 software and visualized using GraphPad Prism 7. The gating was performed according to the uninduced negative control.

### Scanning Electron Microscopy

For SEM, bacteria were fixed with 3% (v/v) glutaraldehyde (Agar Scientific, Wetzlar, Germany) in PBS for at least 4 h, washed in 0.1 M Soerensen’s phosphate buffer (Merck, Darmstadt, Germany) for 15 min, and dehydrated by incubating consecutively in an ascending acetone series (30, 50, 70, 90, and 100%) for 10 min each and the last step thrice. The samples were critical point dried in liquid CO_2_ and then sputter coated (Sputter Coater EM SCD500; Leica, Wetzlar, Germany) with a 10-nm gold/palladium layer. Samples were analyzed using an environmental scanning electron microscope (ESEM XL 30 FEG, FEI, Philips, Eindhoven, Netherlands) with a 10-kV acceleration voltage in a high-vacuum environment.

### Transmission Electron Microscopy

In preparation for TEM analysis, bacteria were fixed with 3% (vol/ vol) glutaraldehyde (Agar Scientific, Wetzlar, Germany) in PBS for at least 4 h, washed in 0.1 M Soerensen’s phosphate buffer (Merck, Darmstadt, Germany), and postfixed in 1% OsO4 in 17% sucrose buffer. After fixation, bacteria were embedded in 2.5% agarose (Sigma, Steinheim, Germany), then rinsed in 17% sucrose buffer and deionized water and dehydrated by ascending ethanol series (30, 50, 70, 90 and 100%) for 10 min each. The last step was repeated 3 times. Dehydrated specimens were incubated in propylene oxide (Serva, Heidelberg, Germany) for 30 min, in a mixture of Epon resin (Serva, Heidelberg, Germany) and propylene oxide (1:1) for 1 h and finally in pure Epon for 1 h. Samples were embedded in pure Epon. Epon polymerization was performed at 90°C for 2 h. Ultrathin sections (70–100 nm) were cut by ultramicrotome (Reichert Ultracut S, Leica, Wetzlar, Germany) and picked up on Cu/Rh grids (HR23 Maxtaform, Plano, Wetzlar, Germany). Contrast was enhanced by staining with 0.5% uranyl acetate and 1% lead citrate (both EMS, Munich, Germany). Samples were viewed at an acceleration voltage of 60 kV using a Zeiss Leo 906 (Carl Zeiss, Oberkochen, Germany) transmission electron microscope.

## Data Availability

The microarray data are available in NCBI via GEO record GSE116655.

## Author Contributions

MH and JF conceived the study. MH and MP performed the experiments and analyzed the data. MH and JF wrote the manuscript. All authors reviewed and edited the manuscript.

## Conflict of Interest Statement

The authors declare that the research was conducted in the absence of any commercial or financial relationships that could be construed as a potential conflict of interest.

## Footnotes

1 http://ncbi.nlm.nih.gov/geo
