# Supplementary material for: The MarR-Type Regulator MalR Is Involved in Stress-Responsive Cell Envelope Remodeling in Corynebacterium glutamicum
Source: Front Microbiol. 2019 May 21;10:1039. doi: 10.3389/fmicb.2019.01039 (PMC6536590; doi:10.3389/fmicb.2019.01039)
Supplement: Supplementary file 1 [file Data_Sheet_1.pdf]

Supplementary Material

1 Supplementary Data

2 Supplementary Figures and Tables

2.1 Supplementary Figures

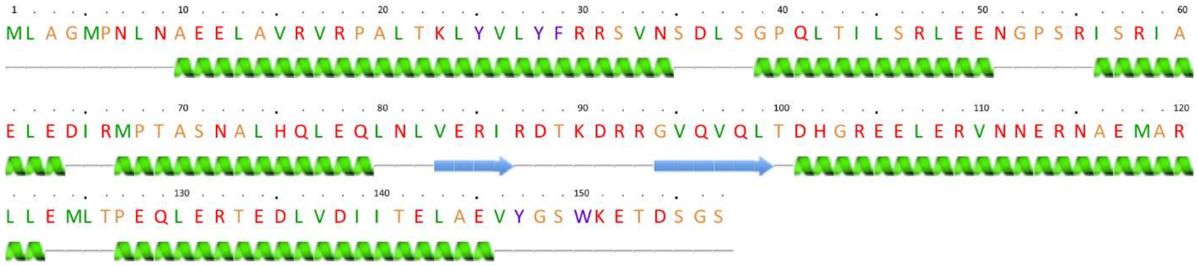

Figure S1: Prediction of the secondary structure of MalR. Using the online tool Phyre[2], the secondary structure of MalR was predicted with 99.9% confidence of 92 % of residues (Kelly L.A., Mezulis S., Yates C., Wass M., Sternberg, M. 2015. Nat. Protoc.10:845–858.).

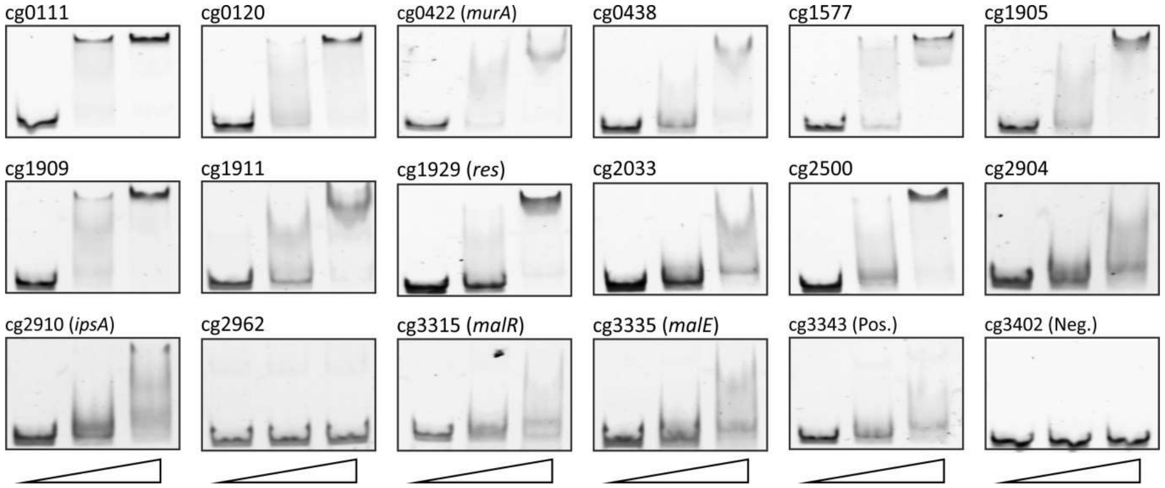

Figure S2: *In vitro* DNA-binding of purified MalR protein. Electrophoretic mobility shift assays (EMSAs) were performed to verify binding of MalR to promoter regions identified via ChAP-Seq. Therefore, MalR was purified with a C-terminal Strep-tag fusion. At total, 90 ng of 100 bp DNA fragments (50 bp up- and downstream the peak maximum) were incubated without protein (first lane), with 3 molar excess (228 nM, second lane) and 10 molar excess of purified MalR (760 nM, third lane) for 30 min in bandshift-buffer (50 mM Tris-HCl, 5 mM MgCl2, 40 mM KCl, 5 % (v/v) glycerol, pH 7.5). Subsequently, samples were separated on a 10% native polyacrylamide gel electrophoresis and gels were stained using SYBR Green I Nucleic Acid Gel Stain (Lonza, Rockland, ME, USA).

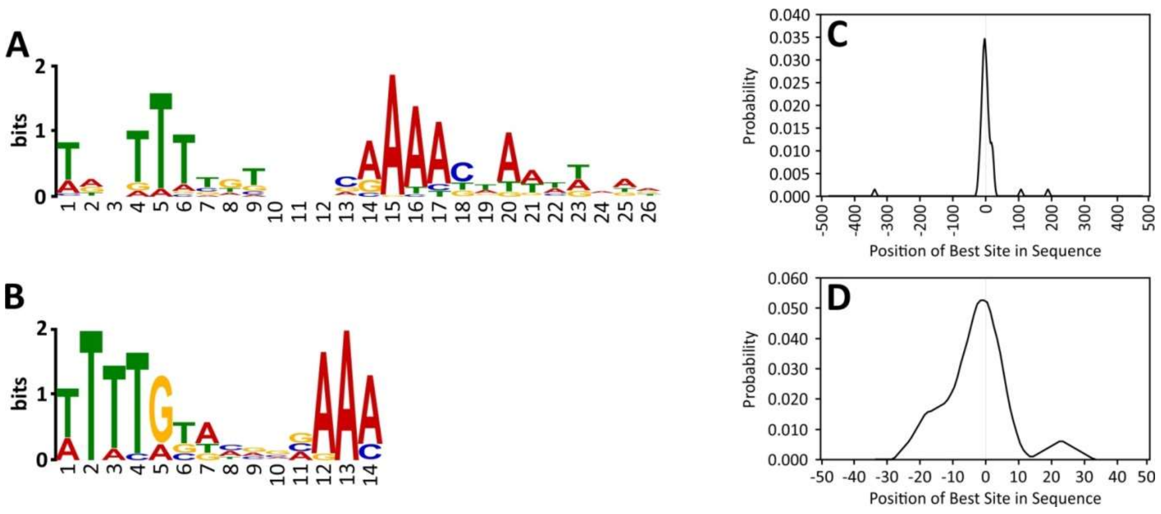

Figure S3: Deduction of a DNA binding motif of MalR The online tool MEME-ChIP (Ma W, Noble WS, Bailey TL. 2014. Nat Protoc 9:1428–1450.) was used to predict MalR binding sites on the basis of all targets sequences (each sequence 1000 bp) identified via ChAP-Seq analysis **(A)** and based on the 16 MalR targets (100 bp each) verified using EMSAs **(B)**. **(C, D)** The distribution of the predicted binding sites throughout the single uploaded DNA sequences was determined using CentriMo (Bailey TL, MacHanick P. 2012. Nucleic Acids Res 40:e128.).

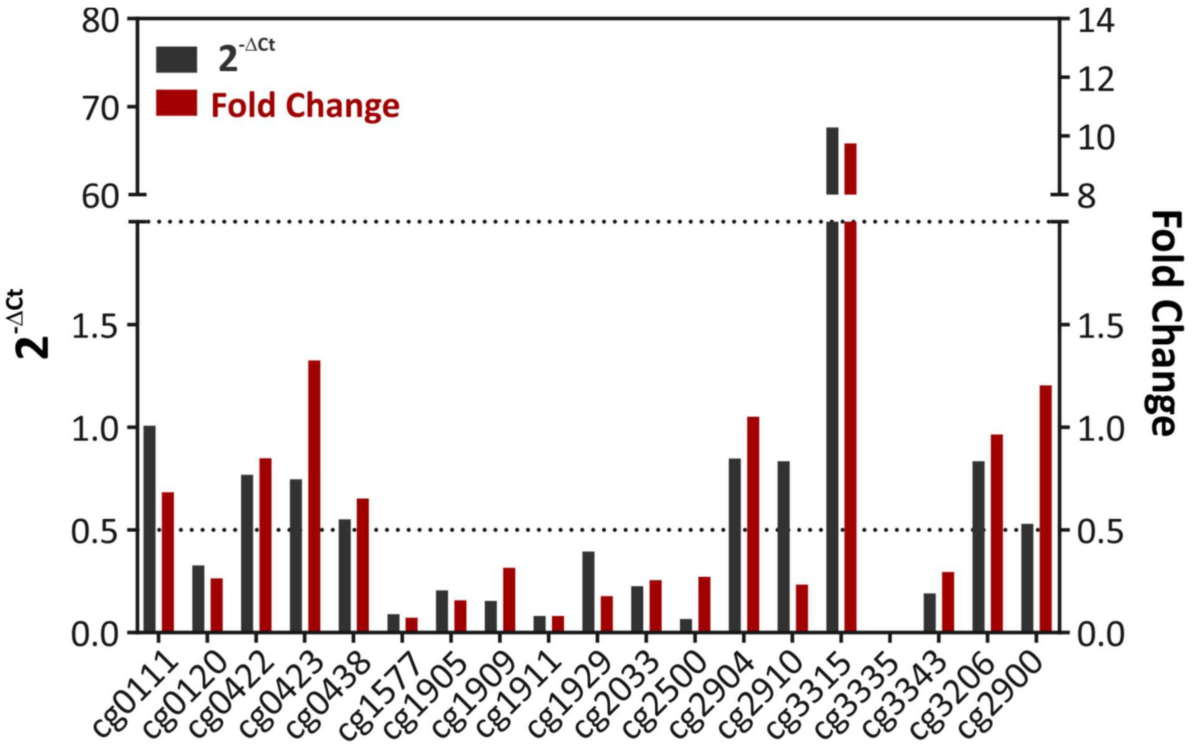

Figure S4: Verification of DNA-microarray data using qRT-PCR. Microarray data were verified with qRT-PCRs (oligonucleotides are listed in Table S3 D). The dark-grey bars represent the relative transcriptional change based on the qRT-PCR data and the red bars show the average fold-change obtained from DNA microarray experiments. The values below 0.5 are classified as “downregulated”, the values above 2 are classified as “upregulated”. In the case of cg3335, the bars are not visible because of very low values (qRT-PCR: 0.018, DNA-microarray: 0.007).

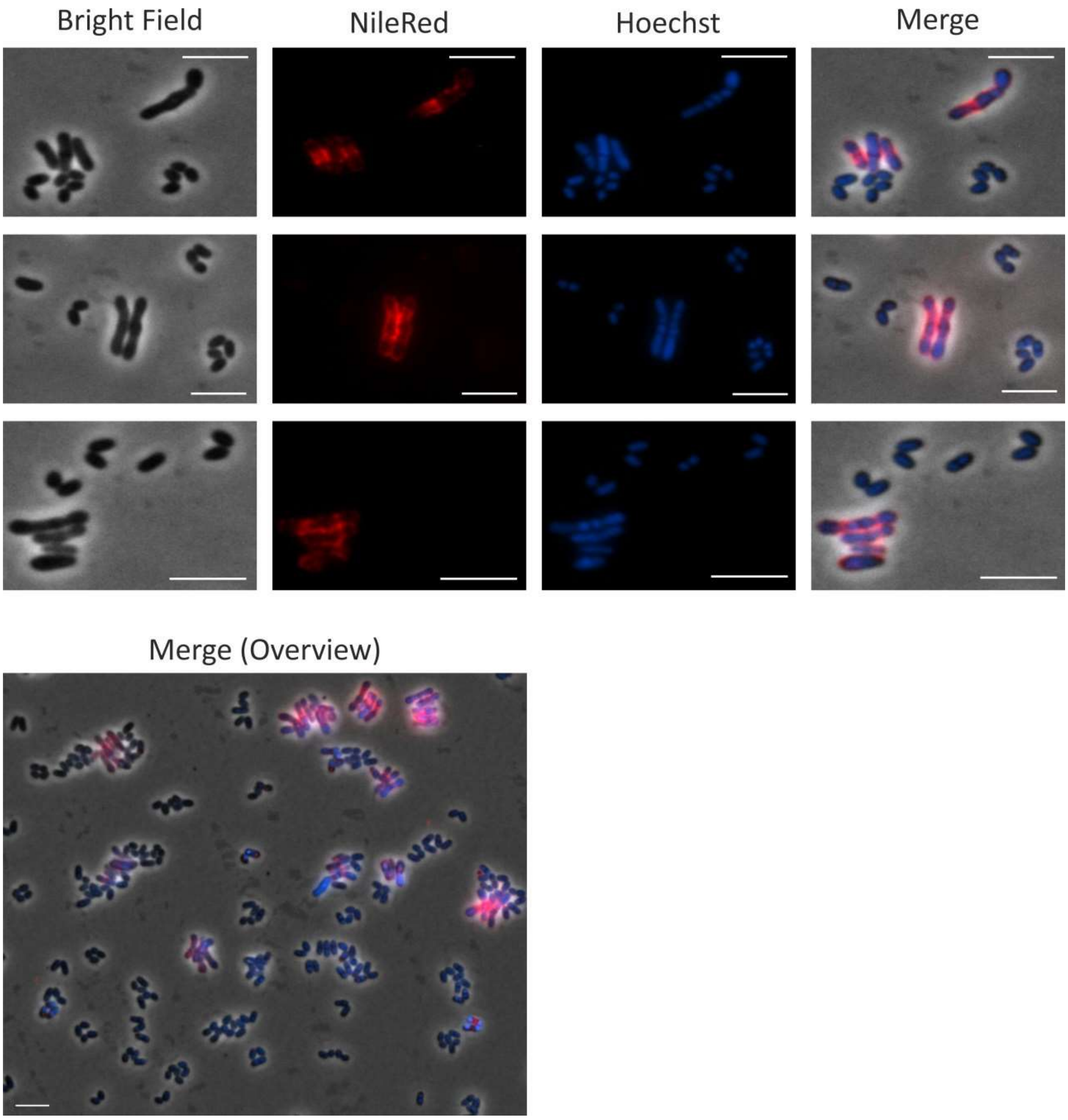

Figure S5: MalR overproduction causes severe growth defects of *C. glutamicum*. For microscopic analysis, cells were grown in CGXII medium for 24 h at 30°C. Shown are *C. glutamicum* ATCC 13032 cells carrying the over expression vector pEKEx2-*malR*. The expression of *malR* is induced by the addition of 100 µM IPTG. Lipid components of the cell membrane were stained with Nile Red (red); DNA was stained with Hoechst 33342 (blue). The white scale bars represent 5 µm. In addition to chosen examples of elongated cells an overview is shown to present the distribution of elongated cells inside the samples.

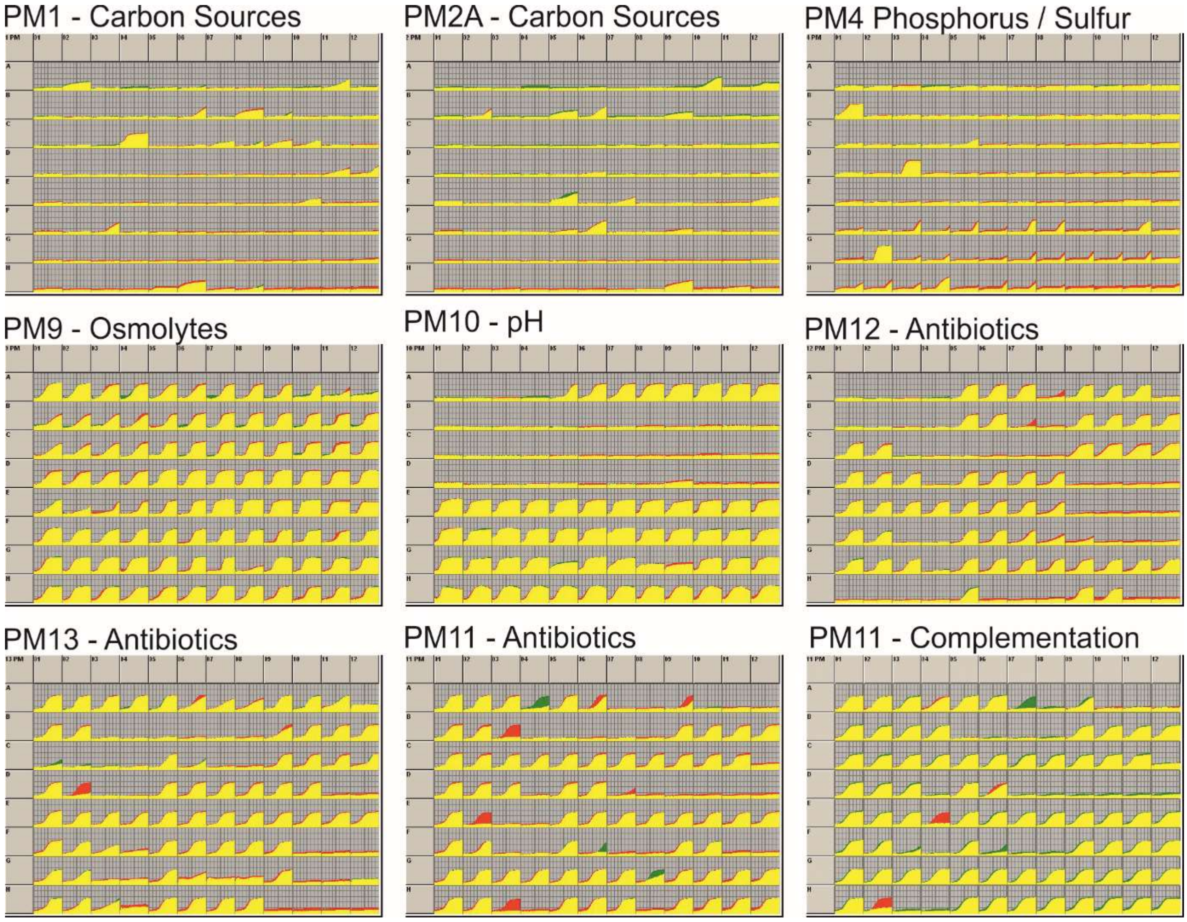

Figure S6: Overview of all tested PM plates for a comparison of *C. glutamicum* Δ*malR* and *C. glutamicum* wild type. An OmniLog System from Biolog (Hayward CA, USA) was used to perform phenotypic microarrays with wild type *C. glutamicum* ATCC 13032 as well as the *malR* deletion strain. The experiments (PM1, PM2A, PM4, PM9, PM10, PM11, PM12, PM13) were conducted like described in the protocol of the manufacturer (Bochner BR, Gadzinski P, Panomitros E. 2001. Genome Res 11:1246–1255.). The wild type strain is displayed in red, whereas the *malR* deficient mutant is green. Yellow means a similar behavior. PM11 was repeated (“PM11 – Complementation”) using the *malR* deficient strain with an empty vector (pEKEx2, green) as well as the *malR* deficient strain carrying the vector pEKEx2-*malR* for complementation studies. The expression of *malR* was induced by the addition of 5 µM IPTG.

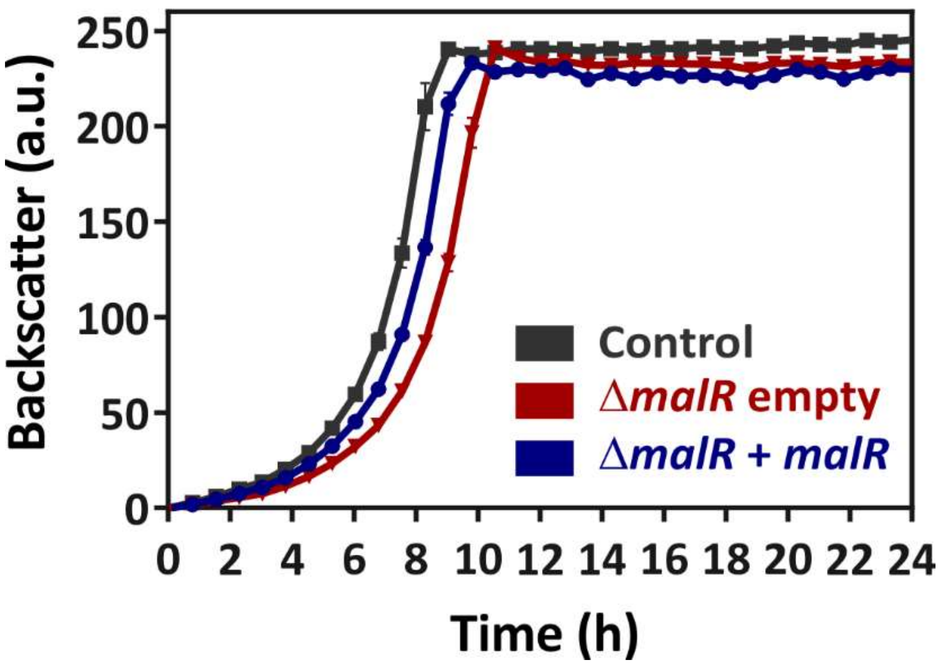

Figure S7: Comparison of the growth of the *C. glutamicum* strains used for the complementation of phenotypes analysed via phenotypic microarrays. To complement the effects of the *malR* deletion, *C. glutamicum* ATCC 13032 wild type cells with the empty vector pEKEx2 (Control) were cultivated in comparison with a strain lacking the *malR* gene either with the empty vector pEKEx2 or with the *malR* encoding vector. The production of MalR was induced using 5 µM IPTG. Remarkably, strain Δ*malR* shows a reduced growth rate under these conditions. This phenotype was successfully complemented by the introduction of plasmid-encoded *malR*. Overall, this finding suggested a slightly increased sensitivity of strain Δ*malR* towards the antibiotic kanamycin.

2.2 Supplementary Tables

Table S3 A: Oligonucleotides used in this study

Number	Sequence (5'-3')
1	AGGTCGACTCTAGAGGATCCTCGAACGGAAATTACTTGGCAATAC
2	CTTCTCGAACTGTGGGTGGGACCAGCTAGCAGAACCGCTGTCGGTCT
3	GCTAGCTGGTCCCACCCACAGTTCGAGAAGTAACAGTTTTCTCCATCTCAACTCC
4	AAACGACGGCCAGTGAATTCACAAGTCCTAGTGGGACGG
5	AGGTCGACTCTAGAGGATCCCCGTCCATTCTTTACCAACTGT
6	CCCATCCACTAAACTTAAACAAGCGGTATTGCCAAGTAATTTCC
7	TGTTTAAGTTTAGTGGATGGGCAGTTTTCTCCATCTCAACTCCG
8	AAACGACGGCCAGTGAATTCGGCACAAGTCCTAGTGGGAC
9	TTTAAGAAGGAGATATACATATGCTGGCAGGCATGC
10	AAATACAGGTTCTCGCTAGCAGAACCGCTGTCGGTCTC
11	AAGCTTGCATGCCTGCAGAAGGAGGAGTCGTATGCCTAATTTAAACGCTGAGGAG
12	AAACGACGGCCAGTGAATTCTTAAGAACCGCTGTCGGTCTC
13	GCCAAGGTAAGTTGTACTTTTTCTG
14	GTAGTTGACGCGGGTGAC
15	TAACAATCCCAACTCGAAGCAC
16	TGACAATCCTCTCCACGAAGC
17	GAGTGTGAGAAACATGGGACGTG
18	ACAGGTTACCAGCCAAAGTG
19	TCGATACCCAGAAAGAATTGCATTTG
20	ATCTCCCTGGATAGTGATCTTGC
21	CCGTCCAGAACTAGGACTATTTG
22	TAATCCCAACAATCGCTTATGACG

23	TTGAAATCGTGATCGCCTGTTATTG
24	AGCCATGTTTGCTTCTCCTTTTC
25	TCAGGTTTACTACCGACTGTAGTAG
26	TTTGAAAGTAGAAATTACTGGTAGTGGATTA
27	TTTACCTTCCTCTACCTAATCTCCC
28	ATCTCAGTAGCGTTGCCTCC
29	TTTCTCATTTCGCCACCCCC
30	CTAAGATGAGTATTTAAGCCCTGTTTAT
31	ACGCCCGTATCGTTTCGC
32	ATGTACTTGTTTAAAACAATAGTTGTCAATG
33	GCACCCATGGTTAGCGTACT
34	TTCTACCGGGGGTAGTAGCG
35	TGTTGCTTCCGCTGACC
36	ACCTTCTATATAAACCTTTTATGAGGGAAATG
37	CTGGACCGAGCTCAATGC
38	TGAAAAATTGTTTTAATATGCAACACAACTA
39	TTGTAGGCGTTGGACACTG
40	AGATTGAAATGATTATGGGTAGGAAAC
41	AAGAGCCGTGATGTTAACAAATG
42	TAGTTAGGTTACACTAATGGTGTCC
43	GGAACAGCACAGAATTAAGGC
44	GAAAAAGCAATAATTTGGACAGAAAAAG
45	AAACCACCCCTGTACAAAATTAGC
46	AAAAACACTAGCTAAATCTGTAGCTCAAC
47	ATGTTTCTTACTTTAAGCGCAGTTAATTG

48	GGATTTAAATTTTGTATTGGAAAGCTAATAATT
49	CATAGGGGTATAGCCTTGAG
50	CAGTGTGCGCAGGTCATGCC
51	GTATGGGTTGAGATTCCGACAG
52	TCTTCCCTATCACCTCCAGTT
53	GGAGCTTTCGCTGACTATCTT
54	AATTCTCCTGCGTCGTCTTT
55	GACGTGGATCGTGTATGGAATTA
56	CAGCCTTCTCAAGGTGGATAAA
57	ACTGTGGATCGACATTCCTTTC
58	CCGCGTCTAAGTCCCTTTAATC
59	ATGACTCGATCCCAGGACTAT
60	TGAATGCGGTATGAGCTAAGG
61	GCCCAAACACCACCGATATT
62	CATCAGTCAAGCAGGTCTGAAC
63	CATCTTCCATCCACAGACCTAAT
64	GAAGTCGGACACGATGTAGAAG
65	ATCTCGTGCTTGCTGTGATTA
66	CTGGGTGAATCCTAAAGACCTG
67	CTGATGGGTCAGTCGTAGTTTC
68	CCGAATAGAGCCAGGAACAAT
69	CTCGACAACGTGAAGCTGTTA
70	CAAGCCAGGTCTGTGTGATT
71	GCTGTAGGTAAGGGCTTTGATA
72	TAGCCAACAGCCTGGTAATC

73	GAGCTTCAGAAACTTGCAACAG
74	CATTGAGGGCGAGGATGATT
75	AGCGAAAGTTCCCGAATCTG
76	CGGACGGTCAGTCTTGTTATG
77	CTCGACACCGCTGAAGATATG
78	CAGTAAGCAGCACTCCGATT
79	GGGTCCACAGCTCACTATTT
80	AGCGGTTGGCATACGAATA
81	AGCAATCAAGGAAGATCCAGAG
82	GATATCGCCAAGGCCAAGAA
83	CCGGTTCTTCTGCATCTTCT
84	GGTTACATCGTCGAAGTCCTTA
85	CAGACTCACAACAACGTCAAAC
86	CTAGTTCGTGGCCAACTTCA
87	TTCCTCACAGATCGCTTTCG
88	GAGCAGGTATGGAGCAACTT

Table S3 B: Construction of plasmids used in this study. Numbers represent oligonucleotide pairs used for PCR (see Table S3 A). The restriction enzymes were used for linearization of the vectors and plasmids were assembled using Gibson assembly. Sequencing for verification of the chromosomal modifications was conducted using primers 13 + 14.

Plasmid	Template	Primers	Vector	Restriction Enzymes
pEKEx2-*malR*	*C. glutamicum* chromosome	11 + 12	pEKEx2	*PstI *EcoRI
pK19-*malR*-C-*strep*	*C. glutamicum* chromosome	1 + 2; 3 + 4	pK19*mobsacB*	*BamHI *EcoRI
pK19-Δ*malR*	*C. glutamicum* chromosome	5 + 6; 7 + 8	pK19*mobsacB*	*BamHI *EcoRI
pET24b-*malR*-C-*strep*	*C. glutamicum* chromosome	9 + 10	pET24b	*NheI *NdeI

Table S3 C: Amplification of EMSAs DNA probes. Indicated are the oligonucleotide pairs used for PCR amplification of the 100 bp fragments. The fragments cover the maximum peak position determined by ChAP-Seq and lay inside the promoter regions of each gene.

Fragment	Gene	Oligonucleotides (Table S1)
1	cg0111	15 + 16
2	cg0120	17 + 18
3	cg0423	19 + 20
4	cg0438	21 + 22
5	cg1577	23 + 24
6	cg1905	25 + 26
7	cg1909	27 + 28
8	cg1911	29 + 30
9	cg1929	31 + 32
10	cg2033	33 + 34
11	cg2500	35 + 36
12	cg2904	37 + 38
13	cg2910	39 + 40
14	cg2962	41 + 42
15	cg3315	43 + 44
16	cg3335	45 + 46
17	cg3343 (positive control)	47 + 48
18	cg3402 (negative control)	49 + 50

Table S3 D: Oligonucleotide combinations for qRT-PCR analysis to verify microarray data. Indicated are the oligonucleotide pairs used for qRT-PCR. Each pair comprises approximately 100 bp fragments and shares similar features regarding GC-content and melting temperature.

Fragment	Gene	Oligonucleotides (Table S1)
1	cg0111	51 + 52
2	cg0120	53 + 54
3	cg0422	55 + 56
4	cg0423	57 + 58
5	cg0438	59 + 60
6	cg1577	61 + 62
7	cg1905	63 + 64
8	cg1909	65 + 66
9	cg1911	67 + 68
10	cg1929	69 + 70
11	cg2033	71 + 72
12	cg2500	73 + 74
13	cg2904	75 + 76
14	cg2910	77 + 78
15	cg3315	79 + 80
16	cg3335	81 + 82
17	cg3343	83 + 84
18	cg3206	85 + 86
19	cg2900	87 + 88

Table S4: Antibiotics affecting the growth of wild type cells or Δ*malR* cells in phenotypic microarrays (BioLog). For evaluation of the sensitivity of the wild type ATCC 13032 or the *malR* deletion mutant, the BioLog plates PM11, PM12 PM13 were used. The table shows antibiotics were both strains showed differences in the metabolic activity (see Figure 6 and Figure S6).

Antibiotic	Better Growth	Substance class
**Gentamicin**	Δ*malR*	Aminoglycoside
**Amikacin**	Δ*malR*	Aminoglycoside
**Lincomycin**	wt	Lincosamide
**Erythromycin**	Δ*malR*	Glycoside
**Chlortetracyclin**	wt	Tetracycline
**Demeclocycline**	wt	Tetracycline
**Tetracyclin**	wt	Tetracycline
**Penimepicyclin**	wt	Tetracycline
**Cefazolin**	wt	β-Lactame; Cephalosporine
**Cephalothin**	wt	β-Lactame; Cephalosporine
**Cefuroxime**	wt	β-Lactame; Cephalosporine
**Amoxicillin**	wt	β-Lactame; Penicilline
